# Supplementary material for: Sedentary Time, Physical Activity, and Sleep Duration: Associations with Body Composition in Fibromyalgia. The Al-Andalus Project
Source: J Clin Med. 2019 Aug 20;8(8):1260. doi: 10.3390/jcm8081260 (PMC6722714; doi:10.3390/jcm8081260)
Supplement: Supplementary file 1 [file jcm-08-01260-s001.pdf]

SUPPLEMENTARY MATERIAL

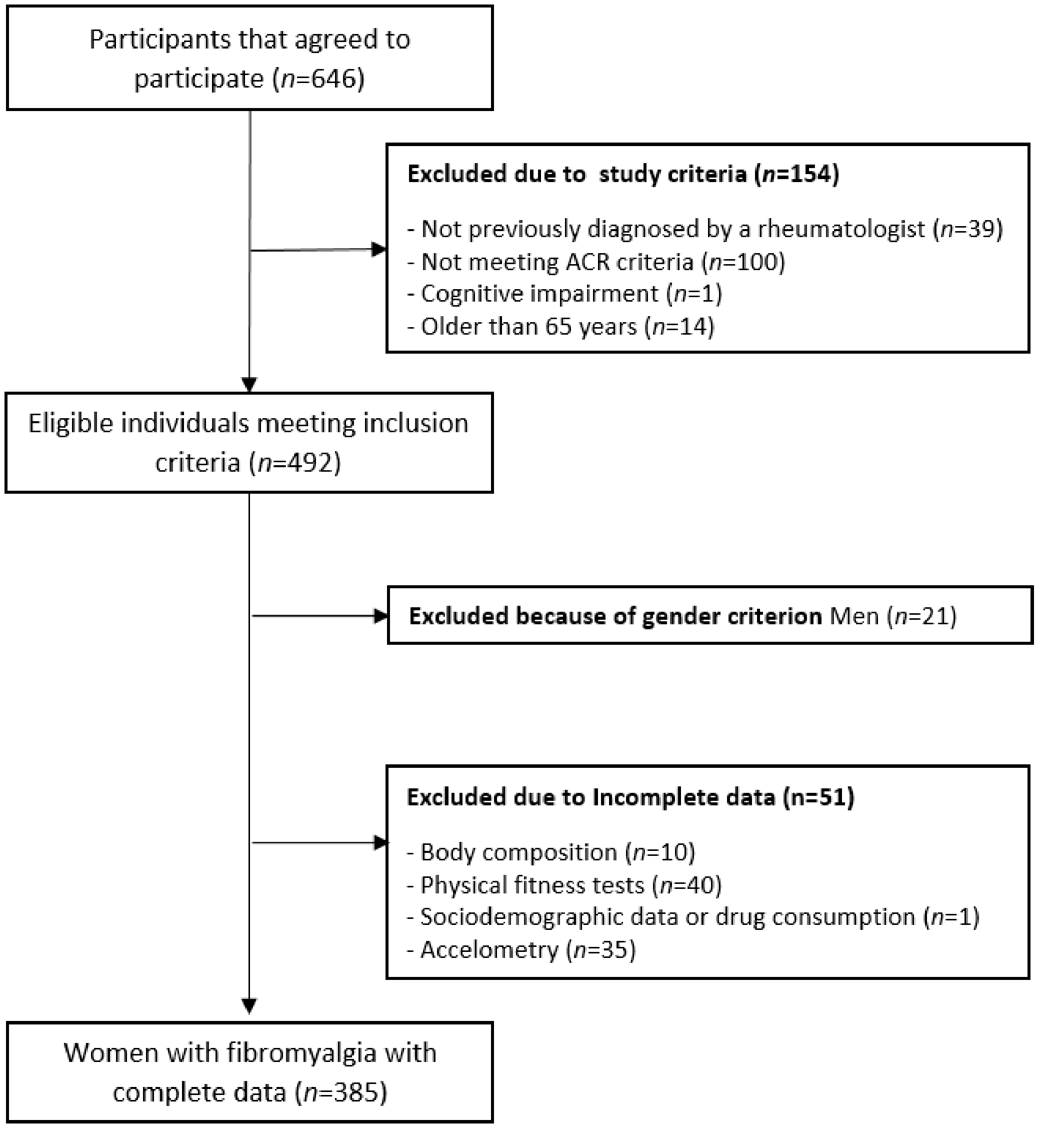

Figure S1. Flowchart of subject enrolment. ACR: American College of Rheumatology.

Table S1. Independent association of sedentary time, light physical activity, bouted moderate-to-vigorous physical activity, sleep duration and physical fitness, with body composition.

	Waist Circumference					Body mass index					Body fat percentage					Muscle mass index [a]				
	B	95 % CI		*β*	Adj. R^2	*B*	95 % CI		*β*	Adj. R^2	*B*	95 % CI		*β*	Adj. R^2	*B*	95 % CI		*β*	Adj. R^2
Sedentary time	0.030***	0.017	0.043	0.24		0.011***	0.005	0.017	0.211		0.013**	0.005	0.021	0.173		0.000	-0.001	0.001	-0.043	
Bouted MVPA[b]	-0.005	-0.017	0.006	-0.047	0.163	-0.003	-0.008	0.002	-0.066	0.117	-0.004	-0.011	0.003	-0.051	0.126	0.000	-0.001	0.001	-0.012	0.380
Sleep duration	0.021**	0.007	0.035	0.144		0.008*	0.002	0.014	0.126		0.01*	0.001	0.018	0.113		0.001	0.000	0.002	0.058	
Sedentary time	0.028 ***	0.015	0.042	0.230		0.010***	0.005	0.016	0.199		0.012**	0.004	0.020	0.162		0.003	-0.001	0.006	0.077	
Bouted MVPA[b]	-0.004	-0.016	0.007	-0.037	0.170	-0.003	-0.008	0.002	-0.054	0.127	-0.003	-0.010	0.004	-0.040	0.135	0.000	-0.003	0.003	-0.005	0.265
Sleep duration	0.022 **	0.009	0.036	0.154		0.008**	0.002	0.014	0.138		0.011*	0.002	0.019	0.125		0.001	-0.002	0.005	0.040	
Physical fitness score	-4.017 *	-7.785	-0.250	-0.107		-1.929*	-3.562	-0.297	-0.121		-2.580*	-4.873	-0.286	-0.115		0.810	-0.120	1.741	0.082	
LPA	-0.033***	-0.047	-0.018	-0.234		-0.012***	-0.019	-0.006	-0.212		-0.014**	-0.023	-0.006	-0.174		0.001	-0.001	0.002	0.044	
Bouted MVPA[b]	-0.012*	-0.023	-0.001	-0.102	0.162	-0.006*	-0.01	-0.001	-0.113	0.118	-0.006	-0.013	0	-0.09	0.127	0.000	-0.001	0.001	-0.002	0.364
Sleep duration	0.021**	0.007	0.035	0.144		0.008*	0.002	0.014	0.127		0.01*	0.001	0.018	0.114		0.001	0.000	0.002	0.058	
LPA	-0.031 ***	-0.046	-0.017	-0.223		-0.012***	-0.018	-0.005	-0.200		-0.013**	-0.022	-0.005	-0.163		-0.003	-0.006	0.001	-0.077	
Bouted MVPA[b]	-0.010	-0.021	0.000	-0.089	0.169	-0.005*	-0.010	0.000	-0.099	0.129	-0.005	-0.012	0.001	-0.076	0.136	-0.001	-0.003	0.002	-0.023	0.265
Sleep duration	0.022 **	0.009	0.036	0.155		0.008**	0.003	0.014	0.139		0.011*	0.002	0.019	0.125		0.002	-0.002	0.005	0.040	
Physical fitness score	-4.019 *	-7.789	-0.249	-0.107		-1.920*	-3.551	-0.289	-0.121		-2.570*	-4.862	-0.277	-0.114		0.811	-0.119	1.741	0.082	

B, non-standardised coefficient; *β*, standardised coefficient; CI, confidence interval; LPA: light physical activity; MVPA: moderate-to-vigorous physical activity. Models were adjusted for accelerometer wear time, age, marital status, professional status, current regular menstruation, and current consumption of alcohol.

[a] Models using muscle mass index were additionally adjusted for fat mass (kg).

[b] MVPA (min/week) accumulated in bouts of at least 10 min.

Significant associations are highlighted in bold with asterisks * P≤0.05, **P≤0.01, *** P≤0.001.
